# CHEMERIN AND FACTORS RELATED TO CARDIOVASCULAR RISK IN CHILDREN AND ADOLESCENTS: A SYSTEMATIC REVIEW

**DOI:** 10.1590/1984-0462/;2018;36;2;00003

**Published:** 2018-01-15

**Authors:** Vanessa Sequeira Fontes, Felipe Silva Neves, Ana Paula Carlos Cândido

**Affiliations:** a Universidade Federal de Juiz de Fora, Juiz de Fora, MG, Brasil.

**Keywords:** Adipokines, Heart diseases, Child, Adolescent, Risk factors, Adipocinas, Doenças cardíacas, Criança, Adolescente, Fatores de risco

## Abstract

**Objective:**

To review findings on chemerin and factors related to cardiovascular risk in children and adolescents.

**Data source:**

A systematic review was performed, according to the standards proposed by the PRISMA guideline, on PubMed, Science Direct, and Lilacs databases. The descriptor “chemerin” was used in combination with “children” and “adolescent”, no time limit applied. The research encompassed only original articles written in English, conducted with human subjects - the adult and elderly populations excluded -, as well as literature reviews, brief communications, letters, and editorials.

**Data synthesis:**

After independent analyses of the studies by two reviewers, seven articles meeting the eligibility criteria, published between 2012 and 2016, remained for the review. Cross-sectional, prospective, cohort, and case-control studies were included. The importance of chemerin adipokines on the risk factors for cardiovascular disease is demonstrated by its association with obesity and diabetes mellitus, as well as clinical, anthropometric, and biochemical parameters. However, the strength of evidence from these studies is relatively low, due to their heterogeneity, with several limitations such as small samples and consequent lack of representativeness, lack of standardization in dosage methods, cross-sectional design of most studies, and impossibility of extrapolating results.

**Conclusions:**

The deregulation of chemerin caused by increased adipose tissue may contribute to the development of cardiovascular diseases, suggesting that this adipokine may play a significant role in early identification of individuals at risk.

## RESUMO

**Objetivo:**

Sintetizar os achados sobre a quemerina e os fatores relacionados ao risco cardiovascular em crianças e adolescentes.

**Fontes de dados:**

Realizou-se uma revisão sistemática de acordo com os itens propostos pela diretriz PRISMA nas bases de dados PubMed, *Science Direct* e Lilacs. Utilizaram-se os descritores *chemerin* de forma associada a *children* e *adolescent*, sem limite de tempo. A pesquisa limitou-se a artigos originais realizados com seres humanos, em língua inglesa, excluindo-se a população adulta e idosa, assim como os artigos de revisão, comunicação breve, cartas e editoriais.

**Síntese dos dados:**

Após análise dos estudos por dois revisores, de forma independente, segundo os critérios de elegibilidade, permaneceram na revisão sete artigos, publicados entre 2012 e 2016. Foram incluídos estudos de delineamento transversal, prospectivo, coorte e caso-controle. A importância da adipocina quemerina nos fatores de risco para doenças cardiovasculares é demonstrada por meio de sua associação com obesidade e diabetes melito, assim como com parâmetros clínicos, antropométricos e bioquímicos. Entretanto, a força da evidência dos estudos é relativamente baixa, em função da heterogeneidade das publicações, destacando-se como limitações o número reduzido das amostras e sua ausência de representatividade, a falta de padronização dos métodos de dosagem, o delineamento transversal de grande parte dos estudos e a impossibilidade de extrapolação dos resultados.

**Conclusões:**

A desregulação da quemerina provocada pelo aumento de tecido adiposo pode contribuir para o aparecimento de doenças cardiovasculares, sugerindo que tal adipocina tem papel relevante na identificação precoce de indivíduos em risco.

## INTRODUCTION

Cardiovascular diseases have been the leading cause of death in Brazil since the 1960s, accounting for two-thirds of all deaths today.^1^ ^,^ ^2^ Cardiovascular risk factors such as overweight, diabetes, systemic arterial hypertension, and dyslipidemias, which used to be more prevalent in adults and the elderly, are now also found in younger individuals.^3^

It is important to stress that the atherosclerotic process onsets in childhood, its severity is proportional to the number of risk factors aggregated, ant it progresses with aging.^4^ Endothelial dysfunction preceding the development of atherosclerosis is associated with raised levels of total cholesterol, low-density lipoprotein (LDL) and triglycerides, insulin resistance, inflammation, and adipokine secretion disorders.^5^ ^,^ ^6^

Adipokines are signaling molecules secreted by the adipose tissue^7^ that function as circulating hormones able to communicate with other organs such as the liver, brain, immune system, and the adipose tissue itself.^8^ ^,^ ^9^ Some adipokines are considered markers of cardiovascular risk, being good methods of diagnosis complementation. Their association with obesity, dyslipidemia, hypertension, and insulin resistance has been pointed out in children and adolescents.^10^ ^,^ ^11^

One of the newly identified adipokines, chemerin, is a chemoattractant protein that plays a role in the differentiation of adipocytes and glucose metabolism.^12^ It is associated with obesity, inflammation, and atherosclerosis,^13^ ^,^ ^14^ and may act in the relationship between increased fat mass and early atherogenic risk in obese children.^13^

Studies on the chemerin adipokine in children and adolescents are newness, but they do show that the concentrations of this adipokine may be altered in different diseases and even in young individuals. As this is a recent discovery presented as a probable marker of cardiovascular risk, the aim of this paper was to conduct a systematic literature review to synthesize the findings about chemerin and cardiovascular risk factors in children and adolescents.

## METHOD

This study was based on the analysis of publications addressing the association of adipokines with cardiovascular risk factors in children and adolescents, being conducted according to the principles of the Preferred Reporting Items for Systematic Reviews and Meta-Analyzes (PRISMA).^15^ The papers were selected after electronic search on MedLine/PubMed (http://www.ncbi.nlm.nih.gov/pubmed/), Science Direct (http://www.sciencedirect.com/), and Lilacs (http://lilacs.bvsalud.org/), with the descriptor “chemerin” in English language, associated with “children” or “adolescent”, indexed by Medical Subject Headings.

The search was conducted in March 2016 simultaneously and independently by two reviewers, according to the databases and predefined search criteria. The research encompassed articles published in English language, as articles written in Portuguese do not appear in such databases. There was no delimitation of year of publication, considering that this adipokine was discovered very recently and the literature lacks studies relating it to the age range of choice.

The inclusion criteria were:

- original articles;
- conducted with humans;
- conducted with children and/or adolescents;
- written in English;
- content related to chemerin and cardiovascular risk factors.

The exclusion criteria were:

- non-original works such as literature reviews, brief communication, letters and editorials;
- samples composed of adults and the elderly;
- samples composed of animal models;
- in-vitro studies;
- articles written in any language other than English;
- articles not addressing to the topic in question.

## RESULTS

The searches conducted in the databases retrieved 180 papers addressing the topic. Initially, a screening for topic-related titles was performed to remove repeated articles and those not meeting the inclusion criteria. Then, the abstracts of the remaining papers were read in detail and publications not meeting the predefined goals for studies were also excluded, totaling 11 studies for full reading after this pre-selection.

**Figure 1:**
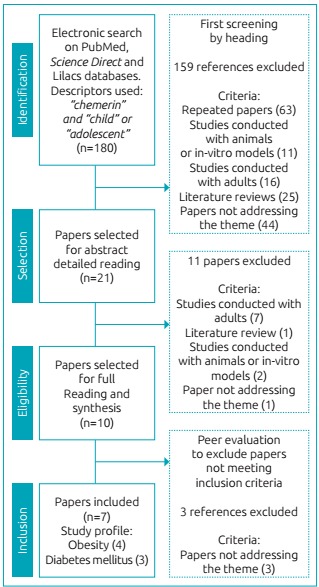
Flowchart of papers’ selection for inclusion in the review.

Then, the papers selected were read in full and summarized. The files were analyzed independently by two evaluators as to inclusion criteria in our review. Discrepant results were reassessed by the examiners. Thus, seven original articles published between 2012 and 2016 (Figure 1) remained in the review and were summed up and organized in Charts 1 and 2 for better understanding.

**Chart 1:** Description of the studies addressing chemerin adipokine and cardiovascular risk factors in children and adolescents included in the systematic review, sorted by author, study country, design, and sample composition.

Reference*	Country	Study design	Sample
Landgraf et al.^13^	Germany	Cohort	Young people aging 7-18 years: obese subjects (n=105) and eutrophic controls (n=69)
Schipper et al.^16^	The Netherlands	Cross-sectional	Young people aging 6-16 years: obese subjects (n=60) and eutrophic controls (n=30)
Verrijn Stuart et al.^9^	The Netherlands	Cross-sectional	Young people aging 6-19 years: type 1 DM of recent onset (n=20), long-term type 1 DM (n=20), healthy controls (n=17)
Redondo et al.^20^	United States	Prospective	Young people aging 2-18 years: obese subjects with type 1 DM of recent onset (n=18) and healthy eutrophic controls (n=30)
El Dayem et al.^17^	Egypt	Transversal	Adolescents aging 14-19 years: type 1 DM for more than 5 years (n=62) and healthy controls (n=30)
Maghsoudi et al.^18^	Iran	Case-control	Female adolescents aging 12-18 years: obese subjects (n=40), and eutrophic controls (n=42)
Maghsoudi et al.^19^	Iran	Case-control	Female adolescents aging 12-18 years: obese subjects (n=38), and eutrophic controls (n=41)

DM: diabetes mellitus. *Papers sorted chronologically.

**Chart 2:** Description of the studies addressing chemerin adipokine and cardiovascular risk factors in children and adolescents included in the systematic review, sorted by author, diagnosis method, chemerin values, and main results.

Reference*	Diagnosis method	Chemerin values	Main results
Landgraf et al.^13^	ELISA	Obese subjects: 117.8±26.4 ng/mL Eutrophic controls: 89.8±16.1 ng/mL	High chemerin levels among obese subjects. Positive correlation between chemerin levels and BMI per age, WHR, leptin, SF, US-CRP circulating white blood cells.
Schipper et al.^16^	Multiplex immunoassay	Obese subjects: 3.0±0.5 µg/mL or 3,000±500 ng/mL Eutrophic controls: 2.8±0.4 µg/mL or 2,800±400 ng/mL	High chemerin levels among obese subjects. Positive correlation between chemerin levels and BMI per age.
Verrijn Stuart et al.^9^	Multiplex immunoassay	Subjects with recent-onset DM: 220 (118-326) ng/mL Subjects with long-term DM: 255 (126-452) ng/mL Healthy controls: 98 (13-256) ng/mL	High chemerin levels among diabetic subjects. No difference in chemerin levels between recent-onset and long-term diabetic subjects.
Redondo et al.^20^	ELISA	Obese subjects with DM: 125.1 (105.8-141.2) ng/mL Healthy controls: 98.4 (79.4-120.0) ng/mL	High chemerin levels among diabetic subjects.
El Dayem et al.^17^	ELISA	Adolescents with DM: 274.44±64.58 ng/mL Healthy controls: 194.42±10.00 ng/mL	High chemerin levels among diabetic adolescents. Positive correlation between chemerin levels and vaspin/LDL-ox.
Maghsoudi et al.^18^	ELISA	Obese female adolescents: 441.8±47.8µg/L or 441.8±47.8 ng/mL Eutrophic controls: 409.3±66.1µg/L or 409.3±66.1 ng/mL	High chemerin levels among female obese adolescents. Negative correlation between chemerin levels and adiponectin, but positive for BMI, WC, HP, WHR, body mass and fat indexes, and BF%. Positive correlation between chemerin levels and US-CRP in female obese adolescents.
Maghsoudi et al.^19^	ELISA	Obese female adolescents: 443.1±47.4 µg/L or 443.1±47.4 ng/mL Eutrophic controls: 408.1±66.5 µg/L or 408.1±66.5 ng/mL	High chemerin levels among female obese adolescents. Negative correlation between chemerin levels and HDL. Positive correlation between chemerin levels and TG, TC, LDL, body mass and fat indexes.

BMI: body mass index; WHR: waist-to-hip ratio; SF: skinfold; US-CRP: ultra-sensitive C-reactive protein; WC: waist circumference; HP: hip perimeter; BF%: body fat percentage; HDL: high-density lipoprotein; TG: triglycerides; TC: total cholesterol; LDL: low-density lipoprotein; LDL-ox: oxidized low-density lipoprotein; DM: diabetes mellitus. *Papers sorted chronologically.

Chart 1 brings information about study site, sample design and composition, while Chart 2 lists diagnosis methods, chemerin levels, and main findings of all seven studies included, in order of publication.

Of all publications included in this review, three are cross-sectional studies,^9^ ^,^ ^16^ ^,^ ^17^ two are case-control studies,^18^ ^,^ ^19^ one is a prospective study^20^, and one is a cohort study.^13^ The studies had been published in several countries and had international samples: Netherlands,^9^ ^,^ ^16^ Iran,^18^ ^,^ ^19^ Germany,^13^ Egypt,^17^ and United States.^20^ After the search with descriptors, no publication from Brazil addressing the topic was found. Four of the studies included were on obesity risk factors^13^ ^,^ ^16^ ^,^ ^18^ ^,^ ^19^ and three on type-1 diabetes mellitus risk factors;^9^ ^,^ ^17^ ^,^ ^20^ four had been conducted with children and adolescents^9^ ^,^ ^13^ ^,^ ^16^ ^,^ ^20^ and three with adolescents only,^17^ ^,^ ^18^ ^,^ ^19^ages ranging from 2 to 19 years.

Chemerin was measured by two different serum dosage techniques: multiplex immunoassay^9^ ^,^ ^16^ and ELISA.^13^ ^,^ ^17^ ^,^ ^18^ ^,^ ^19^ ^,^ ^20^As the concentrations were described in different units of measurement, conversions were performed to make the comparison between works easier. Thus, ng/mL was the measure unit adopted for this study, and chemerin concentrations ranged from 89.8 ± 16.1 ng/mL to 2,800 ± 400 ng/mL in eutrophic subjects; from 117.8 ± 26.4 ng/mL to 3,000 ± 500 ng/mL in obese subjects; and from 125.1 ng/mL (105.8-141.2) to 274.44 ± 64.58 ng/mL in diabetic subjects - widely differing values. The different methods and diagnosis kits for dosages are believed to justify the divergence of values. However, chemerin levels were higher among obese and diabetic subjects compared to controls.

Most studies had evaluated subjects’ inflammatory profile, including other adipokines and proinflammatory cytokines in addition to chemerin.^9^ ^,^ ^16^ ^,^ ^17^ ^,^ ^20^ All papers used anthropometric, clinical, and biochemical variables to identify and categorize nutritional and health statuses of individuals. The most investigated variables were: body mass index (BMI) by age, waist circumference, waist-to-hip ratio (WHR), ultra-sensitive C-reactive protein (US-CRP), total cholesterol, and fractions.

The importance of chemerin adipokine to cardiovascular risk factors is demonstrated by its association with obesity and diabetes, as well as clinical, anthropometric, and biochemical parameters. However, the strength of evidence of studies is relatively low because the methods used vary widely.

The studies selected showed, in addition to higher adipokine values among children and adolescents with obesity and diabetes, an association between WHR, skin folds, waist and hip circumference, percentage of body fat, body fat mass, US-CRP, leptin, vaspin, and white blood cell count. The association was positive and also present with components of the lipid profile: total cholesterol, triglycerides, LDL, and oxidized low-density lipoprotein (LDL-ox). On the other hand, a negative association with high density lipoprotein (HDL) and adiponectin was found.^9^ ^,^ ^13^ ^,^ ^16^ ^,^ ^17^ ^,^ ^18^ ^,^ ^19^ ^,^ ^20^

## DISCUSSION

Although it was first identified in 1997,^21^ chemerin was only recognized as an adipokine in 2007.^22^ So very few studies have addressed adipokine in children and adolescents. Most publications are conducted in the adult population, animal models or mention studies with in-vitro cell cultures. Adult research has shown its role in metabolic syndrome, obesity, diabetes, cardiovascular diseases, Crohn’s disease, arthritis, polycystic ovary syndrome, liver disease, chronic kidney disease, and cancer.^22^ ^,^ ^23^ ^,^ ^24^ ^,^ ^25^ ^,^ ^26^ ^,^ ^27^ As far as our knowledge is concerned, this is the first review written Portuguese that relates this adipokine to cardiovascular risk factors in children and adolescents.

Early identification of risk factors is important to prevent the onset of cardiovascular diseases in adult life; although clinical manifestations of diseases such as stroke and myocardial infarction are common after middle age,^28^ there is evidence that the atherosclerotic process begins in childhood and progresses gradually.^4^

Atherosclerosis has been recognized as an inflammatory disease in which cells of the immune system - such as leukocytes, monocytes, and macrophages - are found in sclerotic lesions.^28^ It is interesting to note that chronic inflammation can be considered a link between the atherosclerotic process and obesity, for adipose tissue is intrinsically involved in the genesis of inflammation. More recent studies have demonstrated that this tissue is not responsible for energy storage only; it is a metabolically active organ with endocrine and paracrine activities that produces numerous substances, adipokines with pro- or anti-inflammatory functions included.^7^ ^,^ ^11^ ^,^ ^29^ ^,^ ^30^

The literature highlights that inflammation, obesity and insulin resistance are a triad, that is, they manifest together and contribute to the development of cardiovascular diseases; in addition, obesity maintenance for prolonged periods is associated with the onset of inflammatory markers.^31^ However, findings indicate that the inflammatory mechanisms that link obesity to metabolic and cardiovascular complications are activated in children and juvenile obesity due to higher concentrations of proinflammatory adipokines in this population when compared to eutrophic children and adolescents.^16^

In this context, we highlight the studies conducted in recent years on chemerin, an adipokine involved in innate and adaptive immune response, firstly codified in its precursor low biological activity form.^32^ ^,^ ^33^ ^,^ ^34^ ^,^ ^35^ Once activated, it triggers rapid defenses in the body by directing dendritic cells and macrophages to injured tissues and inflammation sites.^36^ In adults, chemerin has been associated with metabolic syndrome, obesity, diabetes, and cardiovascular diseases.^23^ ^,^ ^24^ ^,^ ^25^ ^,^ ^26^ ^,^ ^27^ Studies included in this review conducted with children and adolescents demonstrate that serum adipokine concentrations are linked with obesity, diabetes, lipid profile components, and premature vascular inflammation.^9^ ^,^ ^13^ ^,^ ^16^ ^,^ ^17^ ^,^ ^18^ ^,^ ^20^

Chemerin and its receptor CMKLR1 form a complex network involved in the regulation of immune response and can contribute to both the onset and cessation of acute inflammation.^37^ Several mechanisms regulate chemerin signaling, including expression, secretion and processing, and their coordination is essential to determine adipokine levels, localization, and activity.^38^ The chemerin receptors CMKLR1, GPR1 and CCRL2 are well distributed across tissues, and this varied locations may contribute to common and independent chemerin signaling mechanisms and, consequently, its biological functions.^38^

Chemerin has localized action in inflamed or injured tissues. Elevation in levels can directly favor inflammation by recruiting immune system cells. Chemerin also increases expression and secretion of inflammatory mediators to the inflamed spot.^38^ However, there is no consensus as to the involvement of chemerin in the onset or maintenance of inflammatory processes.

In addition to its functions in the immune system, chemerin participates in the regulation of adipocyte metabolism and differentiation, increasing body mass, which may explain its higher concentrations in obese individuals and its association with features related to obesity.^9^ ^,^ ^34^ ^,^ ^39^ ^,^ ^40^ Chemerin signaling is essential during the hyperplasia phase - differentiation of pre-adipocytes into adipocytes.^38^ Increased concentrations of this adipokine in adipose tissue causes the recruitment of immune cells, consequently increasing the expression of inflammatory mediators such as CRP-US, interleukin-6 (IL-6), and tumor necrosis factor alpha (TNF-α).^25^ In terms of cell number, fat storage capacity and endocrine function, the active white adipose tissue is mostly formed in early stages of life, and this is fundamental to shape its pro-inflammatory behavior.^41^

Studies show higher adipokine serum levels in obese adolescents compared to eutrophic subjects.^13^ ^,^ ^16^ ^,^ ^18^ ^,^ ^19^ Landgraf et al.^13^ found about 30% higher concentrations of this adipokine in obese young subjects. Chemerin concentrations are positively correlated with different obesity-related parameters such as BMI per age, waist-to-hip ratio, leptin, and skin folds in children and adolescents.^13^ ^,^ ^16^ Such associations can be explained by the increase in abdominal/visceral adipose tissue, pointed by many authors as a major contribution to chemerin serum levels’ fluctuation.^23^ ^,^ ^42^

In studies conducted only with female adolescent in post-pubertal stage, Maghsoudi et al.^18^ ^,^ ^19^ found that increased abdominal fat was associated with higher adipokine serum levels. Chemerin levels also pair with general and abdominal obesity rates (waist circumference, hip perimeter, waist-to-hip ratio, body fat mass, and body fat percentage)^18^ and with components of the lipid profile (triglycerides, LDL, and total cholesterol).^19^ Although the literature shows no difference between genders as to chemerin serum levels,^13^ ^,^ ^16^ ^,^ ^17^ the fact that male adolescents were not included in samples is a limitation.

One possible explanation for the association of chemerin with the levels of lipid profile components lies in its action on lipid metabolism in the liver, skeletal muscle and adipose tissue, and the stimulation of lipolysis in adipocytes.^19^ ^,^ ^43^ Chemerin is suggested to play a role in the regulation of enzymes responsible for lipid metabolism by reducing the accumulation of adenosine cyclic monophosphate (cAMP) and stimulating calcium release in adipocytes.^19^ Several studies associate the components of the lipid profile with cardiovascular diseases.^21^ ^,^ ^44^ Particularly LDL-ox, which is a lipid peroxidation product, is present in early stages of atherosclerosis.^17^ These particles stimulate adhesion molecules in the endothelium, which initiate the inflammatory process leading to atherosclerosis.^42^ On the other hand, HDL has a protective effect on the endothelium due to its function of reverse cholesterol transport, preventing LDL oxidation of and, thus, reducing its atherogenic potential.^24^ ^,^ ^44^ ^,^ ^45^

Along with elevated serum lipid levels, changes in diabetes such as hyperglycemia and insulin resistance are key to the genesis of cardiovascular diseases. The studies in our sample showed higher chemerin in young people with type 1 diabetes compared to healthy controls. Individuals with recent-onset diabetes also present higher adipokine concentrations.^9^ ^,^ ^17^ ^,^ ^20^ Curiously, an association between chemerin and insulin resistance is observed in both eutrophic and obese young subjects.^16^

Redondo et al.^20^ stated that obese children and adolescents with type 1 diabetes, even recent-onset cases, have a proinflammatory circulating adipokines and cytokines profile that may back up the development of cardiovascular diseases and diabetic complications. The authors found higher chemerin levels in obese children with type I diabetes, as well as in those aging more than 10 years or presenting higher levels of glycated hemoglobin.^20^ Although the mechanisms of action of this adipokine in glucose metabolism have not yet been fully elucidated yet, there seems to be two hypotheses to explain its performance:

1. reduction of insulin-sensitive agents such as transport of glucose type 4 (GLUT-4), leptin, and adiponectin; or
2. increase in levels of insulin-resistant agents such as IL-6.^6^

Increases chemerin levels in young diabetic subjects may be either a compensatory response to insulin resistance or the causal factor of such resistance. The early presence of low inflammation degree and oxidative stress modulated by chemerin causes an acceleration of atherosclerosis.^17^ This adipokine is known to act on glucose metabolism in the liver, skeletal muscle and adipose tissue, promoting regulation of glucose absorption and modulating insulin secretion and sensitivity.^6^ ^,^ ^9^ ^,^ ^34^ Tts role in beta-pancreatic cell homeostasis has also been highlighted.^22^ ^,^ ^46^

Another association found in studies was with the US-CRP, indicating a relationship not only with obesity, but mainly with systemic inflammation.^13^ The inflammatory cytokines released by adipose tissue stimulate the synthesis of C-reactive protein in the liver,^47^ which is observed in inflamed tissues, in atherosclerotic vessels. and in the myocardium after infarction.^28^ In addition, C-reactive protein participates directly in the atherogenesis process and modulates endothelial function.^11^

Although the role of chemerin in inflammation is consensual, there is still no evidence of its actual influence on the process, especially because the literature lacks data on its different isoforms, which assume different functions.^48^ After its secretion, prochemerin undergoes a proteolytic processing, which will determine its activation or deactivation.^49^ Depending on the protease class or cleavage site, chemerin inactive or ­pro/­anti-inflammatory fragments may be produced.^37^ Most of the circulating chemerin is inactive, in the form of prochemerin, and is converted to active when necessary.^50^ The proportion of active and inactive isoforms is determinant for this adipokine’s bioactivity.^38^

Although several studies bring recent findings on chemerin, many are inconclusive and it makes it difficult to understand the actions and functions of this adipokine in the human body. Few publications address the association of chemerin serum levels with cardiovascular risk factors in children and adolescents. This limitation may result from the difficulty in conducting a work with this audience. However, it is pointed out that children and adolescents are not impacted by factors observed in adults, such as smoking, alcohol use, and installed chronic diseases.

As few studies have been conducted on the subject, differences in chemerin levels that have been pointed have not yet been clarified. Some authors suggest that this discrepancy can be attributed to ethnic and environmental diversities or to different methods of sample collection and storage.^39^ Consequently, no reference values to diagnose adipokine alterations in children and adolescents have been proposed so far. The lack of consensus in the literature about reference values for this age group is one of the reasons comparisons between studies is so difficult. However, despite incongruities in dosage method and the absence of reference values, all studies showed higher chemerin values in obese and diabetic patients.

In spite of the difficulty in comparing works, some facts should be given attention. The publications found had heterogeneous samples with reduced subjects’ number and low representativeness, ranging from 50-174 individuals, which makes it impossible to make generalizations and to draw consistent conclusions. Some studies were conducted within a comprehensive age range, such as children and adolescents, without taking into account the differences as to growth, development, and maturation in each phase, which can influence in the presence or absence of cardiovascular risk factors.

Besides these factors, ethnic differences stand out, as the studies were carried out in five different countries from four continents, each of them with a set of population characteristics. Another discrepant item concerns the lack of standardization as to dosage method and unit of measurement used. Different diagnosis methodologies do not allow accurate comparison between studies. Furthermore, commercial kits available for adipokine analysis fail to distinguish active and inactive chemerin isoforms, which sure poses a limitation. The cleavage site by different protease classes is key to chemerin systemic concentrations and biological activity.^51^ ^,^ ^52^ The study design also interferes when it comes to comparison. As most publications are cross-sectional, establishing cause-effect relationships in associations found is impossible. This limitation makes it impossible to extrapolate and generalize the results to other populations.

## CONCLUSION

Studies about chemerin and its association with cardiovascular risk factors are still limited and scarce. The results of this review allow us to conclude that the deregulation of chemerin caused by the increase of adipose tissue may contribute to the onset of cardiovascular diseases, suggesting that this adipokine plays a key role in early identification of individuals at risk.

## References

[1] Barreto SM, Pinheiro AR, Sichieri R, Monteiro CA, Filho MB, Schimidt MI (2005). Analysis of the global strategy on diet, physical activity and
health of the World Health Organization. Epidemiol Serv Saúde.

[2] Guimarães RM, Andrade SS, Machado EL, Bahia CA, Oliveira MM, Jacques FV (2015). Regional differences in cardiovascular mortality transition in
Brazil, 1980 to 2012. Rev Panam Salud Publica.

[3] Molina MC, Faria CP, Montero MP, Cade NV, Mill JG (2010). Cardiovascular risk factors in 7-to-10-year-old children in
Vitória, Espírito Santo State, Brazil. Cad Saúde Pública.

[4] Gazolla FM, Bordallo MA, Madeira IR, Carvalho CN, Collett-Solberg PF, Bordallo AP (2014). Cardiovascular risk factors in obese children. Rev HUPE.

[5] Litwin SE (2014). Childhood Obesity and Adulthood Cardiovascular Disease:
Quantifying the Lifetime Cumulative Burden of Cardiovascular Risk
Factors. J Am Coll Cardiol.

[6] Sypniewska G (2015). Laboratory assessment of cardiometabolic risk in overweight and
obese children. Clin Biochem.

[7] Yamawaki H (2011). Vascular effects of novel adipocytokines focus on vascular
contractility and inflammatory responses. Biol Pharm Bull.

[8] Marreiro DN, Cozzolino SM, Cominetti C (2013). Obesidade: bases bioquímicas e moleculares. Bases bioquímicas e fisiológicas da nutrição: nas diferentes fases da
vida, na saúde e na doença.

[9] Verrijn Stuart AA, Schipper HS, Tasdelen I, Egan DA, Prakken BJ, Kalkhoven E (2012). Altered plasma adipokine levels and in vitro adipocyte
differentiation in pediatric type 1 diabetes. J Clin Endocrinol Metab.

[10] Hung AM, Sundell MB, Egbert P, Siew ED, Shintani A, Ellis CD (2011). A comparison of novel and commonly-used indices of insulin
sensitivity in African American chronic hemodialysis
patients. Clin J Am Soc Nephrol.

[11] Gomes F, Telo DF, Souza HP, Nicolau JC, Halpern A, Serrano CV (2010). Obesity and coronary artery disease: role of vascular
inflammation. Arq Bras Cardiol.

[12] Aydin K, Canpolat U, Akin S, Dural M, Karakaya J, Aytemir K (2015). Chemerin is not associated with subclinical atherosclerosis
markers in prediabetes and diabetes. Anatol J Cardiol.

[13] Landgraf K, Friebe D, Ullrich T, Kratzsch J, Dittrich K, Herberth G (2012). Chemerin as a mediator between obesity and vascular inflammation
in children. J Clin Endocrinol Metab.

[14] Gao X, Mi S, Zhang F, Gong F, Lai Y, Gao F (2011). Association of chemerin mRNA expression in human epicardial
adipose tissue with coronary atherosclerosis. Cardiovasc Diabetol.

[15] Moher D, Liberati A, Tetzlaff J, Altman DG, The PRISMA Group (2009). Preferred reporting items for systematic reviews and
meta-analyses: The PRISMA Statement. BMJ.

[16] Schipper HS, Nuboer R, Prop S, van den Ham HJ, Boer FK, Kesmir C (2012). Systemic inflammation in childhood obesity: circulating
inflammatory mediators and activated CD14++ monocytes. Diabetologia.

[17] El Dayem SM, Battah AA, El Bohy Ael M, El Shehaby A, El Ghaffar EA (2015). Relationship of plasma level of chemerin and vaspin to early
atherosclerotic changes and cardiac autonomic neuropathy in adolescent type
1 diabetic patients. J Pediatr Endocrinol Metab.

[18] Maghsoudi Z, Kelishadi R, Hosseinzadeh-Attar MJ (2015). Association of chemerin levels with anthropometric indexes and
C-reactive protein in obese and non-obese adolescents. ARYA Atheroscler.

[19] Maghsoudi Z, Kelishadi R, Hosseinzadeh-Attar MJ (2016). The comparison of chemerin, adiponectin and lipid profile indices
in obese and non-obese adolescents. Diabetes Metab Syndr.

[20] Redondo MJ, Rodriguez LM, Haymond MW, Hampe CS, Smith EO, Balasubramanyam A (2014). Serum adiposity-induced biomarkers in obese and lean children
with recently diagnosed autoimmune type 1 diabetes. Pediatr Diabetes.

[21] Nagpal S, Patel S, Jacobe H, DiSepio D, Ghosn C, Malhotra M (1997). Tazarotene-induced gene 2 (TIG2), a novel retinoid-responsive
gene in skin. J Invest Dermatol.

[22] Sell H, Laurencikiene J, Taube A, Eckardt K, Cramer A, Horrighs A (2009). Chemerin is a novel adipocyte-derived factor inducing insulin
resistance in primary human skeletal muscle cells. Diabetes.

[23] Bozaoglu K, Bolton K, McMillan J, Zimmet P, Jowett J, Collier G (2007). Chemerin is a novel adipokine associated with obesity and
metabolic syndrome. Endocrinology.

[24] Stejskal D, Karpisek M, Hanulova Z, Svestak M (2008). Chemerin is an independent marker of the metabolic syndrome in a
Caucasian population - a pilot study. Biomed Pap Med Fac Univ Palacky Olomouc Czech Repub.

[25] Lehrke M, Becker A, Greif M, Stark R, Laubender RP, von Ziegler F (2009). Chemerin is associated with markers of inflammation and
components of the metabolic syndrome but does not predict coronary
atherosclerosis. Eur J Endocrinol.

[26] El-Mesallamy HO, El-Derany MO, Hamdy NM (2011). Serum omentin-1 and chemerin levels are interrelated in patients
with Type 2 diabetes mellitus with or without ischaemic heart
disease. Diabet Med.

[27] Fatima SS, Rehman R, Baig M, Khan TA (2014). New roles of the multidimensional adipokine
chemerin. Peptides.

[28] Santos MG, Pegoraro M, Sandrini F, Macuco EC (2008). Risk factors for the development of atherosclerosis in childhood
and adolescence. Arq Bras Cardiol.

[29] Martins LM, Oliveira AR, Cruz KJ, Torres-Leal FL, Marreiro DN (2014). Obesity, inflammation, and insulin resistance. Braz J Pharm Sci.

[30] Tilg H, Moschen AR (2006). Adipocytokines mediators linking adipose tissue, inflammation and
immunity. Nat Rev Immunol.

[31] Silva LR, Stefanello JM, Pizzi J, Timossi LS, Leite N (2012). Atherosclerosis subclinical and inflammatory markers in obese and
nonobese children and adolescents. Rev Bras Epidemiol.

[32] Goralski KB, McCarthy TC, Hanniman EA, Zabel BA, Butcher EC, Parlee SD (2007). Chemerin, a novel adipokine that regulates adipogenesis and
adipocyte metabolism. J Biol Chem.

[33] Bozaoglu K, Curran JE, Stocker CJ, Zaibi MS, Segal D, Konstantopoulos N (2010). Chemerin, a novel adipokine in the regulation of
angiogenesis. J Clin Endocrinol Metab.

[34] Ernst MC, Sinal CJ (2010). Chemerin at the crossroads of inflammation and
obesity. Trends Endocrinol Metab.

[35] Duraiswamy A, Shanmugasundaram D, Sasikumar CS, Cherian KM (2012). Chemerin: a potential target in coronary artery disease - a
review. IJBAR.

[36] Zabel BA, Allen SJ, Kulig P, Allen JA, Cichy J, Handel TM (2005). Chemerin activation by serine proteases of the coagulation,
fibrinolytic, and inflammatory cascades. J Biol Chem.

[37] Mariani F, Roncucci L (2015). Chemerin/chemR23 axis in inflammation onset and
resolution. Inflamm Res.

[38] Rourke JL, Dranse HJ, Sinal CJ (2013). Towards an integrative approach to understanding the role of
chemerin in human health and disease. Obes Rev.

[39] Bozaoglu K, Segal D, Shields KA, Cummings N, Curran JE, Comuzzie AG (2009). Chemerin is associated with metabolic syndrome phenotypes in a
Mexican-American population. J Clin Endocrinol Metab.

[40] Roman AA, Parlee SD, Sinal CJ (2012). Chemerin: a potential endocrine link between obesity and type 2
diabetes. Endocrine.

[41] Shin HY, Lee DC, Chu SH, Jeon JY, Lee MK, Im JA (2012). Chemerin levels are positively correlated with abdominal visceral
fat accumulation. Clin Endocrinol (Oxf).

[42] Barraco GM, Luciano R, Semeraro M, Prieto-Hontoria PL, Manco M (2014). Recently discovered adipokines and cardio-metabolic comorbidities
in childhood obesity. Int J Mol Sci.

[43] Lorincz H, Katkó M, Harangi M, Somodi S, Gaál K, Fülöp P (2014). Strong correlations between circulating chemerin levels and
lipoprotein subfractions in nondiabetic obese and nonobese
subjects. Clin Endocrinol (Oxf).

[44] Rabelo LM (2001). Atherosclerotic risk factors in adolescence. J Pediatr.

[45] Xavier HT, Izar MC, Faria JR, Assad MH, Rocha VZ, Sposito AC (2013). V Diretriz Brasileira de Dislipidemias e Prevenção da
Aterosclerose. Arq Bras Cardiol.

[46] Ernst MC, Issa M, Goralski KB, Sinal CJ (2010). Chemerin exacerbates glucose intolerance in mouse models of
obesity and diabetes. Endocrinology.

[47] Brasil AR, Norton RC, Rossetti MB, Leão E, Mendes RP (2007). C-reactive protein as an indicator of low intensity inflammation
in children and adolescents with and without obesity. J Pediatr (Rio J).

[48] Ferland DJ, Watts SW (2015). Chemerin: A comprehensive review elucidating the need for
cardiovascular research. Pharmacol Res.

[49] Parlee SD, McNeil JO, Muruganandan S, Sinal CJ, Goralski KB (2012). Elastase and tryptase govern TNFa-mediated production of active
chemerin by adipocytes. PLoS One.

[50] Inci S, Aksan G, Dogan P (2016). Chemerin as an independent predictor of cardiovascular event
risk. Ther Adv Endocrinol Metab.

[51] Chang SS, Eisenberg D, Zhao L, Adams C, Leib R, Morser J (2016). Chemerin activation in human obesity. Obesity (Silver Spring).

[52] Han J, Kim SH, Suh YJ, Lim HA, Shin H, Cho SG (2016). Serum chemerin levels are associated with abdominal visceral fat
in type 2 diabetes. J Korean Med Sci.

